# Tamisolve^®^ NxG as an Alternative Non-Toxic Solvent for the Preparation of Porous Poly (Vinylidene Fluoride) Membranes

**DOI:** 10.3390/polym13152579

**Published:** 2021-08-03

**Authors:** Francesca Russo, Tiziana Marino, Francesco Galiano, Lassaad Gzara, Amalia Gordano, Hussam Organji, Alberto Figoli

**Affiliations:** 1 Institute on Membrane Technology (ITM-CNR), Via P. Bucci 17c, 87036 Rende, CS, Italy; f.russo@itm.cnr.it (F.R.); f.galiano@itm.cnr.it (F.G.); a.gordano@itm.cnr.it (A.G.); 2 Center of Excellence in Desalination Technology, King Abdulaziz University, P.O. Box 80200, Jeddah 21589, Saudi Arabia; gzara@kau.edu.sa (L.G.); haorganji@kau.edu.sa (H.O.)

**Keywords:** Tamisolve^®^ NxG, non-toxic solvents, PVDF membrane preparation, phase inversion

## Abstract

Tamisolve^®^ NxG, a well-known non-toxic solvent, was used for poly(vinylidene fluoride) (PVDF) membranes preparation via a non-solvent-induced phase separation (NIPS) procedure with water as a coagulation bath. Preliminary investigations, related to the study of the physical/chemical properties of the solvent, the solubility parameters, the gel transition temperature and the viscosity of the polymer–solvent system, confirmed the power of the solvent to solubilize PVDF polymer for membranes preparation. The role of polyvinylpyrrolidone (PVP) and/or poly(ethylene glycol) (PEG), as pore former agents in the dope solution, was studied along with different polymer concentrations (10 wt%, 15 wt% and 18 wt%). The produced membranes were then characterized in terms of morphology, thickness, porosity, contact angle, atomic force microscopy (AFM) and infrared spectroscopy (ATR-FTIR). Pore size measurements, pore size distribution and water permeability (PWP) tests placed the developed membranes in the ultrafiltration (UF) and microfiltration (MF) range. Finally, PVDF membrane performances were investigated in terms of rejection (%) and permeability recovery ratio (PRR) using methylene blue (MB) in water solution to assess their potential application in separation and purification processes.

## 1. Introduction

On 14 October 2020, the European Commission (EC) divulged a new long-term “Chemicals Strategy for Sustainability” [1], perfectly integrated to the European Green Deal [2], which has been conceived as the guiding principle behind the European chemical industrial innovation. The document relies on sustainability, and in particular on toxic-free environment for protecting people and promoting production innovation towards targeted measures. For achieving this, one of the most successful strategies is to exclude the most harmful chemicals employed for the production with the concomitant boost of substances designed and developed ad hoc to satisfy the safety requirements [3,4,5,6,7]. Food and environmental sectors are primarily involved in the sustainable development and industries are widely adopting eco-friendly technologies to satisfy the balanced growth. Among them, over time, special emphasis has been devoted to membrane technology, which allows to perform separation processes fully respecting the environment integrity and worker health [7,8,9,10,11]. Membranes, both organic and inorganic, are commercially viable and widely used in the most different and disparate sectors, including pharmaceutical and biotechnological fields [8,12,13,14,15]. This is due to the simple preparation methods, uncomplicated processing and good chemical, thermal and mechanical membrane stability. Nevertheless, research is constantly committed to optimize the range of polymeric membranes. In fact, the conventional preparation methods involve the use of organic, harmful compounds; first and foremost, the solvent [16,17,18]. Phase inversion [19,20,21,22], the most versatile and simple technique for membrane fabrication, primarily involves the use of pyrrolidone (NMP), *N*,*N*-dimethylformamide (DMF) and *N*,*N*-dimethylacetamide (DMAC). However, from May 2020, NMP must not be placed on the European Union market, nor used unless stringent conditions are satisfied [23]. NMP is reprotoxic, provokes serious eye, skin and respiratory irritation. These negative properties led to the addiction of NMP in the Registration, Evaluation, Authorisation and Restriction of Chemicals (REACH) restriction list in 2018 [24]. The restriction came into force in May 2020 placing significant limitation on its use. All the limitations geared towards the correct use of the solvent, are finely described in the guideline released by the European Chemical Agency (ECHA) on July 2019 [23], and, as reported in the document, the general approach referred to NMP can be applied to other aprotic solvents similar to NMP (such as DMF and DMAC), if similar REACH restrictions are introduced for other aprotic solvents. In September 2019, European Commission proposed the addition of DMF to the Annex XVII REACH restriction list with a two-year transitional period for all industrial sectors [25], since it is a hepatotoxic and reprotoxic solvent [26]. The planned date for adopting the Annex XVII amendment is the first quarter of 2021 [25]. The temporary period (two years) before the application of the anticipated restriction is intended to guarantee that stakeholders have sufficient time to comply with the proposed restriction and to warrant adequate communication throughout the supply chain. DMAC was labeled by the European Commission as a Substance of very high concern and registered under REACH due to its reproductive toxicity [27]. Bureau REACH of the Netherlands is preparing an Annex XV restriction dossier on the use of DMAC (Expected date of submission: October 2021) [27].

Consequently, NMP, DMF and DMAC producers and user companies face a high level of investment in order to modernize their production processes, by assuring, for example, exhaustive ventilation, limitation for possible exposure time intervals, well-controlled ovens and enameling machines, in addition to appropriate respiratory equipment. Alternatively, the substitution of the toxic organic solvents with less hazardous chemicals might represents a valuable tool, and offers the possibility to investigate new opportunities for the production sector in line with the sustainability principles. During the phase inversion technique, the solvent plays a major role in determining the structure and performances of the final membrane. It is, in fact, a dominant factor for the thermodynamic behavior and for the solvent-nonsolvent kinetic mass transfer. Table 1 shows the most relevant studies on the use of emergent and greener solvents as well as conventional and toxic solvents for PVDF membranes preparation via phase inversion. This table also evidenced the different types of PVDF used in the literature, the preparation conditions and the membrane configuration achieved (such as flat sheet, hollow fibers and/or nanofibers). Emerging organic solvents were explored for membrane production and the obtained preliminary results have shown as their use can really benefit the innovation sector [28]. Among them, methyl-5-(dimethylamino)-2-methyl-5-oxopentanoate (Polarclean) [29,30,31,32,33,34] Cyrene™ [35,36,37,38], dimethyl isosorbide [39], triethyl phosphate [40,41,42,43,44], diethylene glycol monoethyl ether acetate [45], glycerol triacetate (triacetin) [46], acetyl tributyl citrate [47], triethylene glycol diacetate [48], acetyl triethyl citrate [47], triethyl citrate [47] were proposed as non-toxic solvents for poly(vinylidene fluoride) (PVDF) membrane production through phase inversion. Carner et al. [38] reported the use of several greener solvents, including ethyl acetate, 2-methyltetrahydrofuran (2-methylTHF) and Cyrene™ for the preparation of polymer inclusion membranes. In particular, ethyl acetate was chosen as a solvent for dissolving poly(vinylidene fluoride-*co*-hexafluoropropylene) (PVDF-HFP), 2-methylTHF for poly(vinyl chloride) and Cyrene™ for cellulose triacetate. Park et al. [28] described the fabrication of thin film composite (TFC) membranes by employing naturally occurring components, such as recycled poly(ethylene terephthalate) which was used as a support, priamine and tannic acid as monomers, and *p*-cymene and water as solvents. The obtained membranes exhibited promising performance for applications in organic solvent nanofiltration, due to the long-term stability and permeance in six different solvents. Other alternative solvents such as *N*,*N* -dimethyl lactamide (AGNIQUE AMD 3L) [49] were also proposed for PES membranes. In order to overcome the issue related to the use of conventional organic solvents, including their recovery, recycle and/or disposal, Razaliet al. [50] and Paseta et al. [51] described an innovative procedure for obtaining TFC membranes in the total absence of solvent. In fact, polyamide TFC membranes for nanofiltration and reverse osmosis applications were prepared by interfacial polimerization in the vapor phase. Besides phase inversion, membranes in the hollow fiber configuration, can be fabricated also by melt-spinning and stretching without the need for solvents, as recently demonstrated by Ji et al. in 2020 [52].

PVDF represents one of the most required membrane materials, and this is because it offers outstanding properties in terms of thermal, chemical and mechanical stability compared to other commercially available polymers [53,54]. Even though the research on new (preferably bio-based) materials is constantly objective of interest for the achievement of a fully “green” production process, PVDF still remains one of the major versatile thermoplastics applied for ultrafiltration (UF) and microfiltration (MF) separation purposes [53]. Tamisolve^®^ NxG is an organic, polar aprotic solvent, marked by biodegradability and the absence of reprotoxicity [55]. For membrane preparation, Tamisolve^®^ NxG appears as a suitable candidate for the replacement of commonly used toxic solvents in membrane preparation, since it is fully miscible with water and allows the dissolution of semicrystalline polymers due to its high boiling point (241 °C) [56]. Doyen cited Tamisolve^®^ NxG as NMP alternative for making film-supported membranes by using both amorphous and semicrystalline polymers [57]. Bu described methods for manufacturing polyimides films with Tamisolve^®^ NxG instead of NMP [58]. Table 1 summarizes the studies reported in literature for PVDF membranes prepared with more sustainable solvents (including all the works with Tamisolve^®^ NxG) and with traditional solvents. Marino et al. employed Tamisolve^®^ NxG for the first time for preparing PVDF-HFP membranes via vapor induced phase separation (VIPS) tested in direct contact membrane distillation; experiments conducted at different temperatures highlighted performance similar to those of commercially available membranes in polypropylene [56]. Crystallization experiments were investigated with the same PVDF-HFP membranes using Tamisolve^®^ NxG as a solvent via non-solvent phase separation (NIPS) by Saidi et al. [59]. Tamisolve^®^ NxG was also selected for producing a support nanofiltration (NF) membrane for crosslinked PVDF [60] and for NF membranes preparation via spray-modified non-solvent induced phase separation (s-NIPS) [61]. Another important study with Tamisolve^®^ NxG was conducted by Bagnato et al. [62]. They prepared more sustainable PEEK-WC catalytic membranes doped with Ruthenium (Ru) via the VIPS/NIPS technique for the application in the hydrogenation reaction of bio-oil model compounds. In this work, PVDF flat sheet membranes were prepared via Non-solvent-Induced Phase Separation (NIPS) by using Tamisolve^®^ NxG as solvent and water as non-solvent. Polyvinylpyrrolidone (PVP) e polyethylene glycol (PEG) were chosen as pore former additives and the influence of their concentration was studied. The obtained membranes were characterized in terms of morphology, pore size, porosity, thickness, contact angle, AFM and ATFR-IR. Filtration tests were also carried out in terms of pure water permeability (PWP), methylene blue (MB) rejection and permeability recovery ratio (PRR) for potential MF/UF applications in separation and purification processes.

## 2. Materials and Methods

### 2.1. Materials

The PVDF Solef^®^6010 (Solvay Specialty Polymers, Bollate, Italy; Molecular weight Mw = 322 kg/mol) homopolymer was kindly provided by Solvay Specialty Polymers (Bollate, Italy) while Tamisolve^®^ NxG solvent was kindly supplied by Taminco (Gent, Belgium), a subsidiary of Eastman Chemical Company and used without any further purification. Polyethylene glycol-PEG_200_- (Mw = 0.2 kg/mol) and Methylene Blue (MB) hydrate were purchased from Sigma Aldrich (Milan, Italy). Polyvinylpyrrolidone (PVP K17) (Mw = 9 kg/mol) was purchased by BASF (Lud-wigshafen, Germany). The PVDF polymer and PVPK17 were desiccated under vacuum at 40 °C for 12 h before use. Distillate water was used at 15 °C for the coagulation bath and at 60 °C for washing treatment.

### 2.2. Membrane Preparation

The dope solutions were prepared by adding the correct amounts of polymer powder into Tamisolve^®^ NxG solvent and varying the quantities of additives (PVP K17 at 5 wt% and PEG_200_ at 10, 15, 20 and 40 wt%). The concentration of PVDF 6010 polymer was also modified from 10 wt% to 18 wt%. The solutions were maintained under stirring at 80 °C for two hours, until became homogeneous. Subsequently, the dope solutions were kept at same temperature without stirring for 6 h (degassing time) for removing possible air bubbles. The temperature of the solutions at high content of polymer (18 wt%) and additives (PEG_200_: 40 wt%) was fixed at 120 °C. The homogenous solution was cast via the NIPS technique by making use of a glass plate and a manual casting knife with reservoir (Elcometer 3700/1 Doctor Blade, Germany). The knife gap was 350 µm and humidity room was 40 Rh%. The nascent membrane was directly immersed in a coagulation bath of water at 15 °C. The membranes were also washed three times in hot water (60 °C) and then dried in an oven overnight at 40 °C. The casting solution conditions of the PVDF 6010 membranes preparation are listed in Table 2.

### 2.3. Membrane Characterization

#### 2.3.1. Phase Diagram

In order to determine the sol-gel transition temperature for the PVDF 6010/Tamisolve^®^ NxG system, the tube tilting method was utilized. For each concentration (10 wt%, 15 wt%, 20 wt% and 25 wt%), the transparent solutions were prepared at 80 °C. Then, the temperature was decreased by 5 °C every one hour stepwise. The solution was extracted from the oil bath for a time lag of 10 s every time the temperature changed until the gel phase was achieved.

#### 2.3.2. Scanning Electron Microscopy (SEM)

The morphology was determined by Scanning Electron Microscopy (SEM; Zeiss EVO, MA100, Assing, Italy). The cross-section samples were fractured in liquid nitrogen and fitted vertically on a sample holder. All samples (top, bottom, cross sections) were coated with gold by sputter machine (Quorum Q 150R S).

#### 2.3.3. Viscosity

The viscosity of solutions was measured by using a rotational rheometer (Brookfield, Synchro-Lectric viscometer model: LV). The temperature used was 80 °C.

#### 2.3.4. Pore Size and Pore Size Distribution

The pore size and pore distribution measurements were carried out by using the wet-up/dry-up method with a PMI Capillary Flow Porometer (CFP1500 AEXL, Porous Materials Inc., Ithaca, NY, USA) connected to the software Capwin. Each membrane sample was soaked in the wetting fluorinate liquid Fluorinert FC-40 for 5 h. Two measurements were carried out for each membrane and average and the standard deviation were calculated.

#### 2.3.5. Thickness, Porosity and Contact Angle

Membrane thickness was detected by a digital micrometer (Carl Mahr, Göttingen Germany) and registered in eight regions of each membrane. Porosity values of membranes were calculated during 24 h, using the gravimetric method and considering the weight of membrane samples before and after the immersion in Kerosene. The porosity was determinate by following equation:

(1) $$\varepsilon (\%)=\left\{\frac{\left(\mathrm{Ww}-\mathrm{Wd}\right)\;/\rho k}{\left(\mathrm{Ww}-\mathrm{Wd}\right)/\rho k+\left(\frac{\mathrm{Wd}}{\rho P}\right)}\right\}\times 100$$

This equation includes the information about the weight of the wet (W_w_) and dry membrane (W_d_), the density of Kerosene (ρ_k_ = 0.81 g/cm^3^) and the density of PVDF polymer (ρ_P_ = 1.78 g/cm^3^). The measure was performed on three different pieces of the same membrane and the average percentage and standard deviation were calculated.

The wettability of the membranes was analyzed by means of a CAM 200 contact angle instrument (KSV Instruments LTD, Helsinki, Finland) using ultrapure water droplets (5 µL). For each membrane, five measurements were acquired and averages and standard deviations were calculated.

#### 2.3.6. Atomic Force Microscopy (AFM)

The surface roughness of the membranes was investigated by using atomic force microscopy (AFM) from Nanoscope IIIA (Digital Instruments, VEECO Metrology Group). The AFM pictures were detected in tapping mode (velocity: 2.54 Hz) with the dimension of 5 µm × 5 µm and the roughness parameters were quantified in term of root-mean-square roughness (Rq) and mean roughness (Ra). One hundred values were taken in different parts of each membrane and the average and error have been reported.

#### 2.3.7. Attenuated Total Reflectance Fourier Transform Infrared Spectroscopy (ATR-FTIR)

The different phases (α, β, γ) of PVDF were analyzed by ATR-FTIR spectra at a resolution of 4 cm^1^. The measurements were carried out in different points of the sample area at the same pressure with a micrometer torque (UATR crystal Diamond/ZnSe—Spectrum One System by Perkin Elmer Instruments).

#### 2.3.8. Water Permeability Test and Filtration Experiments

The water permeability (PWP) of the membranes was evaluated using a cross-flow cell system (DeltaES.r.l., Rende, Italy) connected to the feed by a gear pump (Tuthill Pump Co., California). Two samples for each membrane were investigated and the average and standard deviation were calculated. The values were measured using the following equation:

(2) $$\mathrm{PWP}=\frac{Q}{\left(A\;\cdot \;t\;\cdot \;\Delta P\right)}$$

where Q is the permeate volume (L), A is the membrane area (m^2^), t is the time (h), and p is the pressure (bar). The area of the membrane was 8 cm^2^, the measurements were evaluated after a stabilization time of 40 min (time required to reach the steady state condition) at a transmembrane pressure of 2 bar and at room temperature (25 °C). After the stabilization period, the water permeate was collected within 60 s after applying three different transmembrane pressures (the stabilization period of one to another was about 10 min).

The filtration experiments were performed with the same cross-flow filtration set-up used for water permeability tests at 25 °C under pressures ranging from 2 to 3 bar depending on the membrane water permeability. An aqueous methylene blue (MB) solution (10 mg/L) was used as a feed and filtered through the membranes. Membrane samples were conditioned in the MB solution overnight before being tested. The rejection (R) of the membranes towards MB was calculated by the Equation (3):

(3) $$R\%=\left(1-\frac{\mathrm{Cp}}{\mathrm{Cf}}\;\right)\times 100$$

where Cp is the concentration of MB in the feed and Cp is the concentration of MB in the permeate. The concentration of MB was determined via spectrophotometer (ShimadzuUV-160A, Kyoto, Japan) at a wavelength of 664 nm. The flux of MB was estimated by Equation (2). After the rejection analysis of MB, the membrane samples were washed with water for 1 h and the pure water flux of these cleaned membranes was re-measured. Then, the permeability recovery ratio (PRR) was also calculated applying Equation (4):

(4) $$\mathrm{PRR}\;(\%)=\left(\;\frac{\mathrm{Pwp}2}{\mathrm{Pwp}1}\;\right)\cdot \;100$$

where Pwp2 is the water permeability of the cleaned membrane and the Pwp1 is the initial pure water permeability.

## 3. Results and Discussion

### 3.1. Phase Separation of PVDF /Tamisolve^®^ NxG Systems

**Figure 1:**
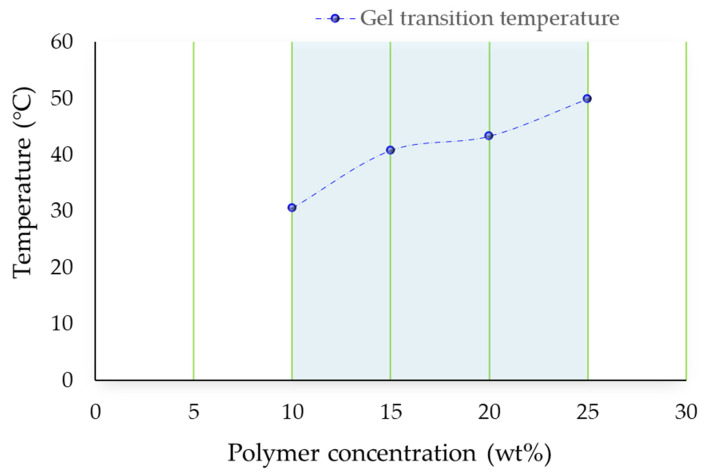
Gel transition phase of PVDF polymer.

In order to investigate the thermodynamics of membrane preparations and the interaction between polymer, solvent and non-solvent, Hansen solubility parameters (HSP) were considered. HSP can be an important tool to discuss the influence of different components during phase inversion and on membrane structure and/or performance. According to Hansen theory, the three-dimensional solubility parameters (HSP) in terms of dispersion (δd), polar (δp), and hydrogen bonds (δh) for the solvent Tamisolve^®^ NxG are 17.8 MPa ^½^, 8.2 MPa ^½^, 5.9 MPa ^½^, respectively. The total solubility parameter of the solvent is δ_T_: 20.3 MPa ^½^ [39] that is very close to the one of PVDF (δ_T_: 23.2 MPa ^½^) [71] and very far to the one of water (δ_T_: 47.8 MPa ^½^) being the non-solvent of the system. Figure 1 shows the sol-gel transition temperature of PVDF/Tamisolve^®^ NxG system as a function of polymer concentration (10 wt%, 15 wt%, 20 wt% and 25 wt%). The sol-gel transition is the temperature at which the solution becomes gel and the solution flow is not observed. The results confirmed that the sol-gel transition temperature increased as the PVDF concentration increased. This outcome is in accordance with literature data which indicate the necessity to use higher temperatures to keep polymer–solvent dope solutions in a homogeneous state when the polymer concentration increases.

### 3.2. Morphology and Viscosity

Membrane morphology can be influenced by the polymer/solvent/non-solvent system during the phase inversion process, by the viscosity of the dope solution and also by the operative conditions. Tamisolve^®^ NxG represents the next-generation of polar aprotic solvents [72] with a high safety profile and solubility parameters comparable to traditional solvents such as NMP, DMF, DMA, indicating the possibility to produce membranes by the NIPS procedure. The different structures of PVDF membranes can be obtained by varying the concentration of polymer (10 wt%, 15 wt% and 18 wt%) and additives (PVPK17 and PEG200) in the dope solutions.

**Figure 2:**
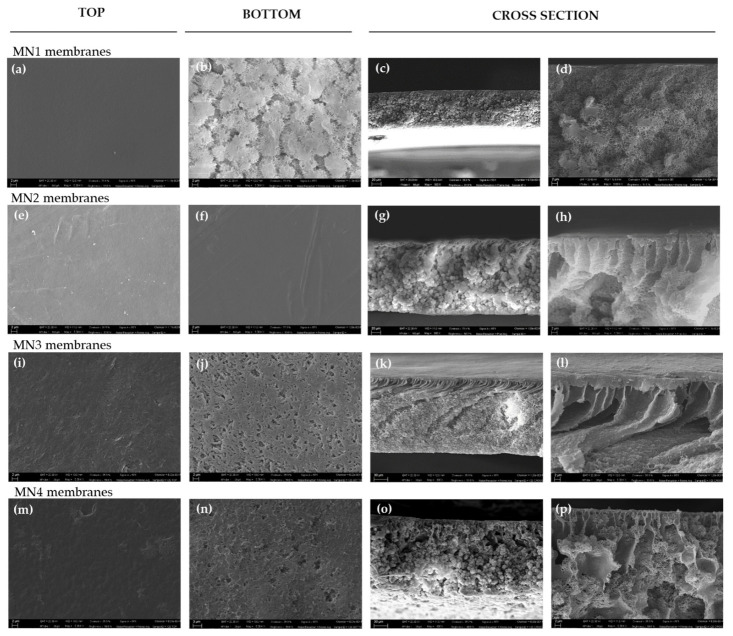
SEM pictures of prepared PVDF (15 wt%) membranes: (**a**–**d**) surface (top and bottom Mag. 5.00 KX) and cross-sections (Mag. 800 X and 5.00 KX) of the MN1, prepared without any additive; (**e**–**h**) surface (top and bottom Mag. 5.00 KX) and cross-sections (Mag. 800 X and 5.00 KX) of the MN2, prepared with PVP K17 (5 wt%); (**i**–**l**) surface (top and bottom Mag. 5.00 KX) and cross-sections (Mag. 800 X and 5.00 KX) of the MN3, prepared with PEG 200 (20 wt%) and (**m**–**p**) surface (top and bottom Mag. 5.00 KX) and cross-sections (Mag. 800 X and 5.00 KX) of the MN4, prepared with PEG 200 (40 wt%).

Figure 2 shows the morphology of the top, bottom and cross-section of the membranes prepared at 15 wt% of PVDF polymer from the polymer–solvent system (MN1) and from the polymer-additive-solvent system (MN2, MN3 and MN4) where just one of the two additives (PVP K17 and PEG 200) was used.

The top surface of the membranes (Figure 2a,e) was characterized by a dense and compact layer, in agreement to what is generally observed for the membranes prepared by the NIPS procedure [34,40].

The formation of this top layer is the result of the fast de-mixing rate of the cast dope solution which enters in contact with the water coagulation bath. At the interface between the casting solution and the coagulation bath, the increase in polymer concentration leads to a decrease in the surface porosity and to the formation of the dense top structure [73]. The top surface of the MN3 membrane (Figure 2i) is characterized by the presence of pores owing to the use of PEG200 (20 wt%) employed as a pore forming agent in the casting solution. The bottom surface of MN1 membranes (Figure 2b) showed, on the contrary, a porous and flatted structure. The reason lies in the fact that the solvent/non-solvent exchange is slowed down by the formation of the dense top layer and the creation of a more porous structure is hence favored.

The cross-section of MN1 (Figure 2c,d) is characterized by a dense skin layer and a spongy sublayer with some visible spherulitic structures whose formation can be the result of a polymer crystallization effect as reported by Zhang et al. [74], Chuang et al. [75] and Ali et al. [76]. They have pointed out that the formation of the sublayer is strongly influenced by the precipitation rate of the top layer that can cause a reduction of solvent outflow rate towards the coagulation bath retarding the de-mixing process. This is in accordance with the low time of the membrane formation in the coagulation bath (~8 min for MN1) as reported in Table 2. A delayed de-mixing is generally responsible for the formation of membranes characterized by a dense surface with a porous sublayer [77]. The addition of PVPK17 and PEG 200 in the dope solution played an important role in the de-mixing rate during the phase inversion and affected the final morphology of the membranes. These additives are employed to improve the membrane pore dimension and porosity thanks to their affinity with the water contained in the coagulation bath. Their hydrophilic nature can, in fact, induce a much faster de-mixing leading to the formation of finger-like macro-void structures as showed in the cross-sections of the MN2 (Figure 2g,h), MN3 (Figure 2k,l) and MN4 (Figure 2o,p) membranes. For MN2, the presence of only PVP at 5 wt% resulted in a membrane characterized by spherulites along the cross-section (Figure 2g) and by a dense top surface (Figure 2e). Besides that, the bottom surface of M2 membrane (Figure 2f) is totally dense respect to the bottom surface of MN1 (Figure 2b) when only PVDF and Tamisolve^®^ NxG were used. This could be ascribed to the properties of the PVPK17, that, as well reported in the literature [66], tends to intertwine with PVDF chains due to its high affinity for the polymer. In this view, the solvent outflow during the solidification phase of the polymeric membrane can be responsible of the formation of a dense bottom surface [78,79]. However, the more open structures were evidenced when PEG 200 was added to the dope solution at 20 wt% (MN3 membrane). The presence of PEG 200 resulted in a finger-like structure with a sponge-like sublayer (Figure 2k,l) and a porous bottom side (Figure 2j). This can be attributed to the role that PEG 200 plays in the mechanism of phase inversion. Wang et al. [80] observed that PEG 200 (from 0 wt% to 20 wt% of content) promoted the transit from a delayed de-mixing (sponge like structure for MN1) to instantaneous de-mixing (finger-like structure for MN3). On the other hand, the presence of additives can increase the viscosity of the dope solution (Table 3) which can limit the solvent/non-solvent exchange rate and can promote the formation of more open structures. The viscosity increased from 375.5 ± 1 cP for MN1 (without additives) to 815.2 ± 1 cP for MN2 (with PVP K17) and to 787.3 ± 1 1 cP for MN3 (with PEG200).

**Figure 3:**
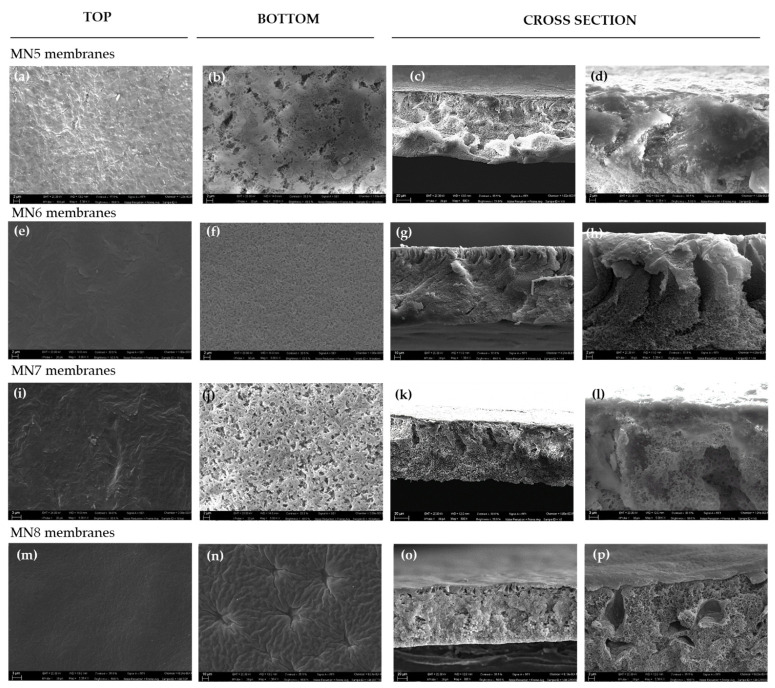
SEM images of membranes prepared with constant concentrations of PVDF (15 wt%) and PVPK17 (5 wt%) and different concentrations of PEG 200: (**a**–**d**) surface (top and bottom Mag. 5.00 KX) and cross-section (Mag. 800 X and 5.00 KX) of MN5; (**e**–**h**) surface (top and bottom Mag. 5.00 KX) and cross-section (Mag. 800 X and 5.00 KX) of the MN6; (**i**–**l**) surface (top and bottom Mag. 5.00 KX) and cross-section (Mag. 800 X and 5.00 KX) of MN7; and (**m**,**o**,**p**) surface (top and bottom Mag. 5.00 KX), (**n**) surface (bottom Mag. 1.00 KX) and cross-section (Mag. 800 X and 5.00 KX) of MN8.

The effect of PEG 200 was also evaluated by keeping constant the concentration of the polymer (15 wt%) and of the additive PVP (5 wt%). In this case, the PEG 200 concentration was varied from 10 to 40 wt % (membranes MN5- MN8) and their morphology is reported in Figure 3.

It was clearly observed that all membranes showed a dense top layer (Figure 3a,e,i,o) and a porous bottom layer (Figure 3b,f,j,n). No relevant differences were found for the cross-section structures from 10 wt% (dynamic viscosity of 951 ± 1 cP) to 20 wt% (dynamic viscosity of 1546 ± 1 cP) of PEG 200. The main difference is observed for the membrane prepared with the highest concentration of PEG 200 (MN8) whose bottom layer (Figure 3n) appeared less porous and more compact. In this case, the higher dope solution viscosity could be responsible for a slower polymer precipitation with a reduction of solvent and additives diffusion during the phase inversion.

The effects of higher and lower polymer concentrations (18 wt% and 10 wt%) on membranes morphology were also evaluated while keeping a constant concentration of PEG 200 (20 wt%) and varying the concentration of PVP (0 and 5 wt%) (membranes MN9-MN12).

**Figure 4:**
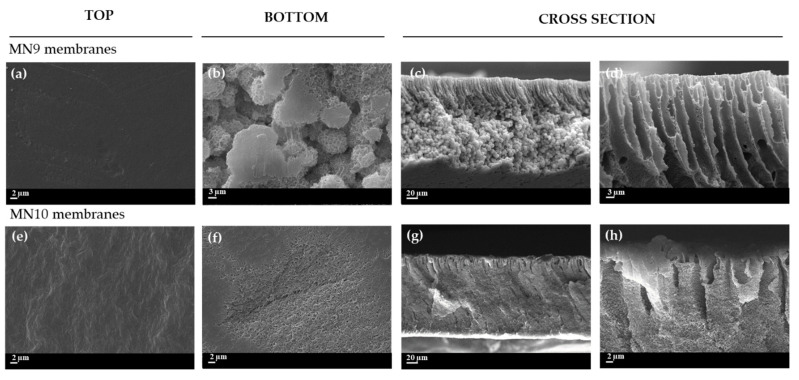
SEM image of prepared PVDF (18 wt%) membranes: (**a**–**d**) surface (top and bottom Mag. 5.00 KX) and cross-section (Mag. 800 X and 5.00 KX) of MN9 with PEG_200_ (20 wt%) and (**e**–**h**) surface (top and bottom Mag. 5.00 KX) and cross-section (Mag. 800 X and 5.00 KX) of MN10 with PEG_200_ (20 wt%) and PVP K17 (5 wt%).

**Figure 5:**
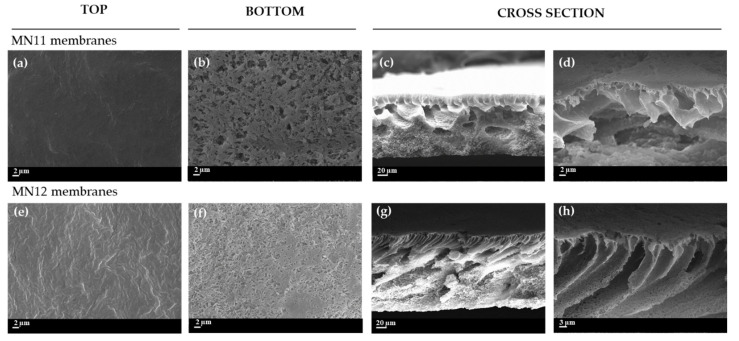
SEM image of prepared PVDF (10 wt%) membranes: (**a**–**d**) surface (top and bottom Mag. 5.00 KX) and cross-section (Mag. 800 X and 5.00 KX) of MN11 with PEG_200_ (20 wt%) and (**e**–**h**) surface (top and bottom Mag. 5.00 KX) and cross-section (Mag. 800 X and 5.00 KX) of MN12 with PEG_200_ (20 wt%) and PVP K17 (5 wt%).

In Figure 4, the structure of the MN9 and MN10 membranes, prepared, respectively, using 18 wt% of PVDF without and with PVP, is reported. Both membranes exhibited dense surfaces (Figure 4a,e and Figure 5a,e). MN9 clearly presented a finger-like structure in the upper part of the membrane similar to the one observed for the analogue membrane M3 prepared with 15 wt% of polymer. The sublayer of the membrane is characterized by a spherulitic structure. However, the addition of PEG 200 (MN10) resulted in a fading of the fingers and in the suppression of the spherulitic structure in favor of a more compact morphology. This can be related to the increase in viscosity as a consequence of the PVP addition which could have hindered the mobility of PVDF 6010 polymer chains [56].

The morphology of MN11 and MN12 membranes, prepared at lower polymer content (10 wt%), is reported in Figure 5. It is possible to observe that the bottom of both membranes showed a porous surface with MN11 surface (Figure 5b) more open than MN12 (Figure 5f). The cross-section for MN11 exhibited a more porous structure, with the presence of macro-voids (Figure 5c,d). Generally, at low content of polymer, the diffusion of the non-solvent into the system (water in this case) is high, ensuring the formation of very porous structures [81]. This was also confirmed by the values of dynamic viscosity (675 ± 1 cP) for MN11 membrane reported in Table 3. By adding the PVP K17 in the same solution (MN12), the macro-voids became longer and the top layer appeared clearly finger-like with a sponge-like structure on the sublayer (Figure 5g,h).

### 3.3. Pore Size and Pore Size Distribution

**Figure 6:**
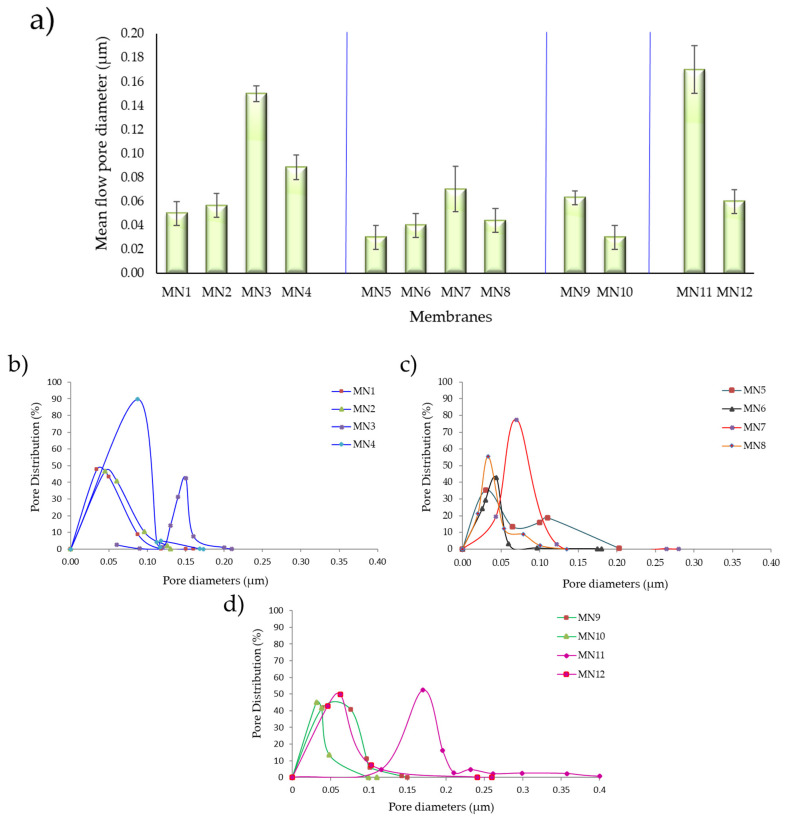
(**a**) Mean flow pore diameter and (**b**–**d**) pore size distribution of investigated membranes.

The pore size values and pore distribution of the PVDF 6010 membranes are investigated and reported in Figure 6a–d. It is possible to observe in Figure 6a that the pore dimension is between the ultrafiltration (UF) and microfiltration (MF) range, from 0.03 µm to 0.17 µm. The differences found agree with SEM analysis and depend on the types and content of additives and on polymer concentration. For MN1, at 15 wt% of polymer concentration and without the presence of additives, the pore size was 0.05 µm. No significant difference was observed when the PVP K17 (5 wt%) was added in the dope solution (0.06 µm for MN2). These values are in agreement with the results of pore size reported in literature for PVDF membranes prepared via NIPS procedure [39,82,83] and with the pore distribution reported in Figure 6b. An important increase in pore size is observed with the addition of PEG 200 (20 wt% for MN3) due to its ability to act as a pore former, while for higher concentrations (40 wt%) the pore size decreased (0.09 µm for MN4). As reported in Figure 6b, the pore distribution for MN4 is very narrow with a 90% of pores at 0.09 µm. When both additives were used for membranes preparation, the increase in PEG 200 concentration (10 wt%, 15 wt%, 20 wt%) led to an increase of the pores dimension as visible for MN5, MN6 and MN7 which showed a pore size of 0.03 µm, 0.04 µm and 0.07 µm, respectively. However, the further increase of PEG 200 to 40 wt% (MN8) led to a slight decrease in pore size (0.05 μm) as also occurred in MN4 membrane. As argued in SEM discussion, the increase in the solution viscosity, probably delayed the formation of the membrane leading to a more compact structure. This result was also confirmed by the pore size distribution, shown in Figure 6c, that was not very uniform for MN5 and MN8 membranes. The MN9 and MN10 membranes, prepared with a higher concentration of polymer (18 wt%) and in the presence of PEG 200 (20 wt%) and PVP K17 (only for MN10), exhibited values of pore size of 0.06 µm and 0.03 µm, respectively. In this case, in agreement with SEM pictures and pore size distribution reported in Figure 6d, the presence of PVP resulted in a more dense structure with a reduced pore size as a consequence of a solution viscosity increase. By decreasing the polymer content (10 wt%), the pore size increased, with a value of 0.17 µm for MN11 and 0.06 µm for MN12 with a narrow pore distribution (Figure 6d), in comparison to the membranes prepared at higher polymer concentrations (MN9 and MN10). In this case, the non-solvent water can penetrate into the casting film with a considerable velocity and can diffuse into the system ensuring the formation of larger pores.

### 3.4. Thickness, Porosity and Contact Angle

Thickness, porosity and contact angle results are summarized in Table 4. The thickness of PVDF membranes prepared via NIPS using Tamisolve^®^ NxG as a solvent, ranged from 90 ± 1 µm to 138 ± 1 µm depending on the dope solution composition. For MN1 membrane at 15 wt% of PVDF, the thickness was 100 ± 1 µm which slightly increased to 125 ± 1 µm for MN2 with the presence of PVP K17.

The MN3 membrane, containing the 20 wt% of PEG 200, exhibited a thickness of 104 ± 1 µm and when its concentration increased from 20 wt% to 40 wt% (MN4) the thickness slightly increased with a value of 107 ± 1 µm. The thickness for the membranes containing both PVP and PEG 200 (MN5, MN6, MN7 and MN8) ranged from 109 ± 1 µm to 123 ± 1 µm. It was also observed that the higher polymer concentration (18 wt%) led to an increase in the thickness with values of 133 ± 1 µm for MN9 and 138 ± 1 for MN10. On the contrary, the thickness decreased at lower polymer concentration (10 wt%) for MN11 and MN12 (90 ± 1 µm and 96 ± 1 µm, respectively). These results are consistent with the porosity values measured. The porosity is between 71% and 89% for all the membranes prepared. As expected, the porosity decreased with the increase of polymer concentration as a consequence of the denser nature of the membranes. The same results were observed for the membranes at high PEG 200 content. By measuring the wettability of the prepared membranes, the MN1 membrane, not containing any additive, was the more hydrophobic membrane with a CA of 91°. The lower values of contact angle, observed for the other membranes, can be related to the addition of the two hydrophilic additives PVP K17 and PEG 200 into the casting solution. The contact angle of these membranes ranged from 77 ± 1° to 88 ± 1° for top side and from 78 ± 1° to 110 ± 1° for the bottom side.

The different values of CA for the bottom surfaces might be related to the different roughness of this side (more porous as shown in SEM pictures) respect to the top layer. According to the Cassie–Baxter model, in fact, porous surfaces generally show higher apparent CA values due to their ability to entrap air pockets within their pores [84].

MN1 membrane presented the highest value of CA (91°) in line with the hydrophobic nature of the polymer. The lower values observed for the top layers of the other membranes can be the consequence of the presence of the hydrophilic pore formers (PVP and PEG) which could not be totally washed away from the membranes [44].

### 3.5. AFM Analysis

**Figure 7:**
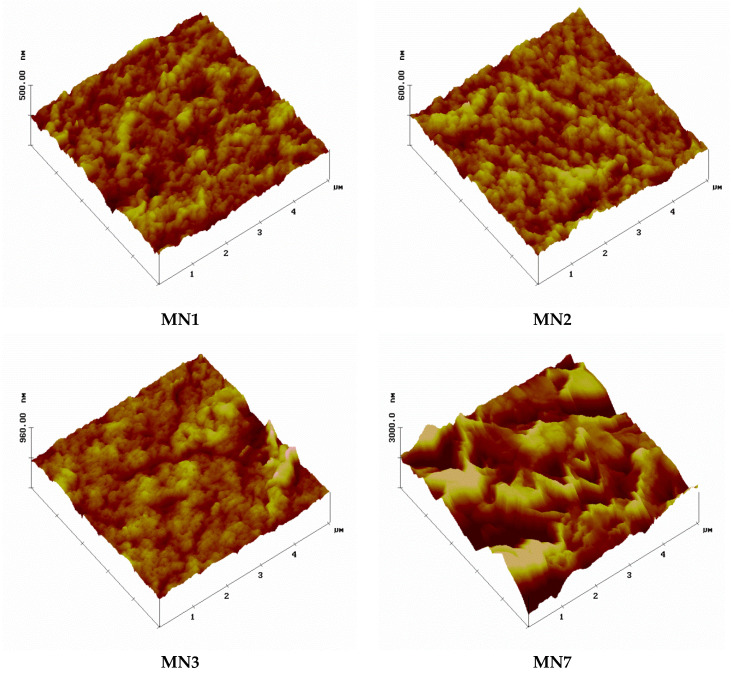
AFM three-dimensional surface structure (roughness) of the prepared PVDF membranes (MN1, MN2, MN3 and MN7).

**Figure 8:**
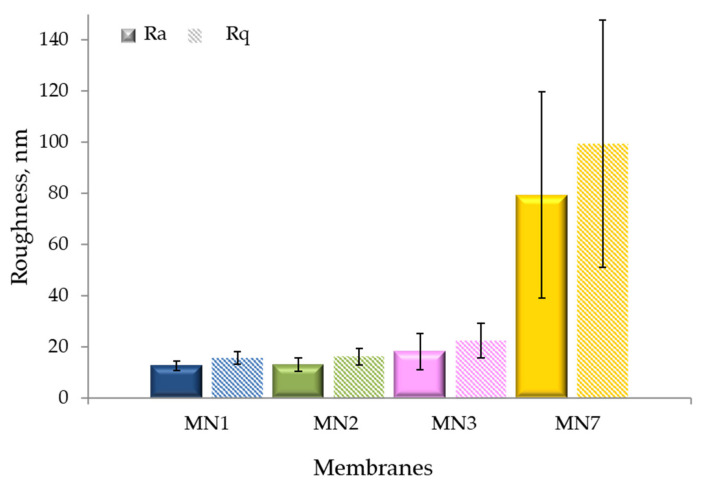
Surface roughness parameters for the prepared PVDF membranes (MN1, MN2, MN3 and MN7).

The AFM three-dimensional structures and surface parameters, in terms of root-mean-square roughness (Rq) and mean roughness (Ra), are reported in Figure 7 and Figure 8, respectively. The AFM analysis were conducted for the MN1 membrane without the presence of additives and for the MN2, MN3 and MN7 membranes prepared at different concentrations of additives as shown in Table 2. As already observed in the SEM pictures (Figure 2), MN1 and MN2 membranes showed a smooth surface as confirmed by the lower values of average roughness. The Rq value was, in fact, 16 ± 3 nm for both MN1 and MN2 membranes while the Ra values were 12 ± 2 nm and 13 ± 3 nm for MN1 MN2 membranes, respectively. The AFM pictures for MN3 membranes, prepared with 20 wt% of PEG200, confirmed the presence of a porous structure on the top surface in accordance with the SEM analysis with slightly higher values of roughness (Rq = 22 ± 7 nm, Ra = 18 ± 7 nm). The surface roughness parameters were much higher for the MN7 membrane (Rq: 99 ± 48 nm, Ra: 79 ± 40 nm) probably due to the addition of both PVPK17 and PEG200 in the dope solution which promoted the formation of a more porous structure (as shown in porosity and pore size data).

### 3.6. ATR-FTIR Spectra

**Figure 9:**
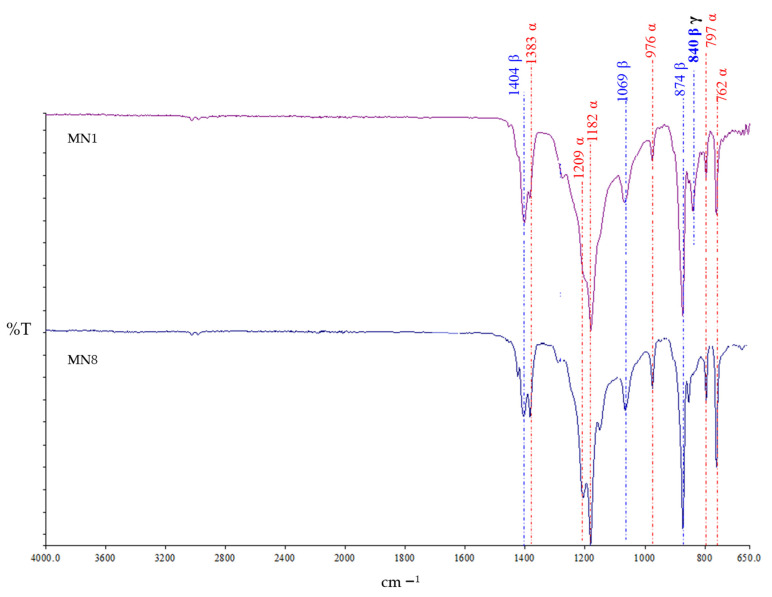
ATR-FTIR spectra highlighting α and β phases of the MN1 and MN8 membranes prepared without additives and with additives (PVP K17 and PEG 200), respectively.

To verify the crystalline polymorphism of PVDF membranes, the infrared spectroscopy (ATR-FTIR) analysis was carried out and the spectra are reported in Figure 9.

As reported in the literature [85,86,87], PVDF polymer has five crystalline polymorphs: α (phase II), β (phase I), γ (phase III), δ and ε. The α phase represents a non-polar form, kinetically more stable and dominant in the presence of non-polar solvents, whereas the β-phase is a highly polar form, which is thermodynamically more stable and can be induced by the presence of additives and/or operation conditions. The polar form can be individuated for the γ-phase, but weaker than the β phase due to the presence of a gauche bond in every fourth repeating C–C units [88,89]. The ATR-FTIR peaks identified at 1383, 1209, 1182, 976, 797 and 762 cm^−1^ correspond to the α phase, while the peaks at 1404, 1069, 874 and 840 cm^−1^ confirm the presence of the β phase. The 840 cm^−1^ peak in the MN1 spectrum is common for both β- and γ-phases, as reported in literature [90,91] and it disappeared in the spectrum of the MN8 membrane. Besides polymer properties, the final polymorphism of PVDF membranes can be influenced by the polarity of the solvent employed for its solubilization, by the type of additives present in the dope solution and by the temperature that can influence the crystallization time of PVDF. Generally, PVDF crystals can reorganize following the most stable phase at the employed temperatures. The β phase of PVDF is difficult to be obtained at high temperatures since the polymer chains are more freely to move.

The predominance of the α phase for both membranes indicate that kinetic factors play a major role in membranes in the PVDF crystallization process.

### 3.7. Filtration Properties

#### 3.7.1. Water Permeability Results

**Figure 10:**
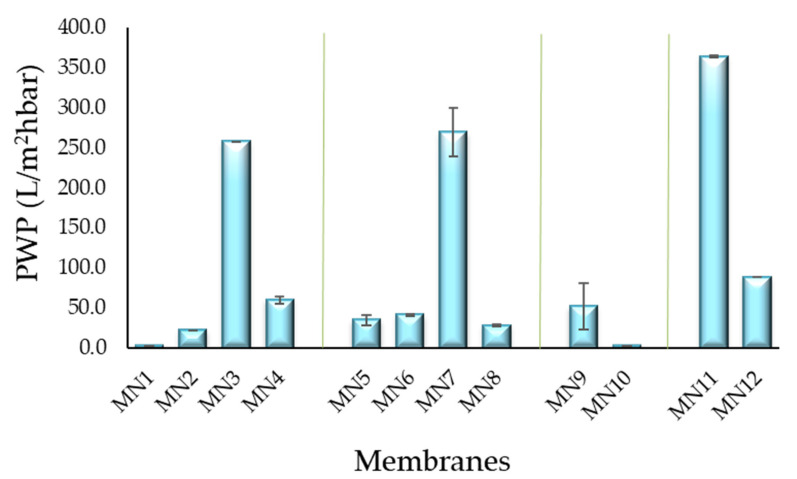
PWP results of investigated membranes.

The water permeability (PWP) values are reported in Figure 10. For the membrane prepared at 15 wt% of PVDF polymer via NIPS without additives (MN1) and with the presence of PVP K17 (MN2), PWP resulted much lower than the membranes prepared with PEG 200. For MN1, no PWP was detected (until 4 bar). This result is in agreement with the literature data for the same type of membranes [39,92]. There is probably no water passage through the membrane due to the presence of a thick dense layer. The values of PWP increased from 22 L/m^2^ h bar for MN2 prepared with 5 wt% of PVP K17 to 258 L/m^2^ h bar for MN3 at 20 wt% of PEG 200. The PWP of all membranes also increased as the PEG concentration increased from 0 wt% (MN1) to 20 wt% (PWP ranged between 51 L/m^2^ h bar and 364 L/m^2^ h bar for MN3, MN7, MN9 and MN11) and declined sharply as the concentration rose from 20 wt% to 40 wt% (60 L/m^2^ h bar for MN4 and 28 L/m^2^ h bar for MN8). This effect can be attributed to the role that PEG plays on the viscosity of the solution and on the hydrophilicity of the membrane. The hydrophilic PEG (until 20 wt%) encourages the instantaneous de-mixing and promotes the formation of finger-like macro-void structures, leading to the enhancement of water permeability of the membrane. However, when the concentration of PEG increases until 40 wt%, the water permeability of the membranes decreases due to the lower porosity and pore size. The PWP of membranes containing both PEG and PVP, at different concentrations of polymer (MN5, MN6, MN7, MN8 at 15 wt%, MN10 at 18 wt% and MN12 at 10 wt%), decreases in accordance to their lower pore size. The PWP of the membrane at 18 wt% of PVDF (MN10) was not detected respect to the corresponding MN9 membrane obtained without the presence of PVP K17 (51 L/m^2^ h bar); while the PWP of the membrane at 10 wt% of PVDF (MN12) showed a PWP of 88 L/m^2^ h bar with respect to MN11 (364 L/m^2^ h bar).

#### 3.7.2. Filtration Results

**Figure 11:**
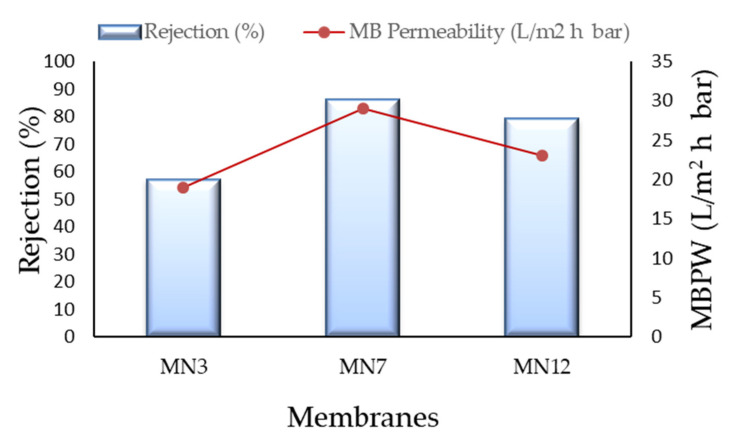
Filtration tests results with MB dye for the investigated membranes.

In order to demonstrate the applicability of prepared PVDF membranes in MF/UF processes, MN3 MN7 and MN12 membranes were tested in filtration experiments using MB dye. The performance of the membranes was investigated in terms of rejection (%) and MB permeability (MBPW), as reported in Figure 11.

The MB rejection for the MN3 was about 56% with a MB permeability of 19 L/m^2^ h bar. The low rejection of this membrane is related to the high pore size of the membrane (about 0.15 μm), in the MF range. The rejection of MN7 was higher (about 86%) with a MBPW of 29 L/m^2^ h bar, while the rejection for the MN12 was 79% with a MBPW of 23 L/m^2^ h bar. The close values of MB rejection and MBPW for these two membranes can be also related to their very similar pore size (about 0.06 μm).

The permeability recovery ratio (PRR) for the PVDF membranes is shown in Table 5. In the same table, the performance of PVDF membranes reported in the literature and prepared with other greener or traditional toxic solvents are also shown. The PRR value for the membranes evaluated in this work ranged from 81 to 94%. The results are comparable with the ones obtained by Ngang et al. [93] for PVDF membranes formed by using DMAc (PRR: 94%). Other greener solvents were used for PVDF membranes and the MB rejection was investigated. In particular, Alyarnezhad et al. [44] prepared PVDF membranes from 13 wt% polymer by using TEP as a green solvent (Table 5) obtaining 53% of rejection. A similar result, in terms of rejection (51%), was obtained by Benhabiles et al. [43] that produced a membrane of blended PVDF/PMMA. In the case of toxic solvents (DMAc, DMF and NMP) the rejection of MB for a series of membranes was about 40–45 wt% [94,95,96] with a very variable water permeability.

## 4. Conclusions

This work demonstrated the possibility of employing Tamisolve^®^ NxG as a non-toxic solvent for the preparation of PVDF 6010 membranes. Two different additives (PVP K17 and PEG 200), as well as different polymer concentrations (10 wt%, 15 wt%, 18 wt%) were investigated. The effect of high content of PEG 200 (from 10 wt% to 40 wt%) was also studied. The results confirmed the possibility to obtain asymmetric structures with different morphology (sponge or finger-like macro-void) with a wide range of pore size. Thickness, porosity, contact angle and water permeability were also influenced by dope solution composition. The MN1 membrane prepared at 15 wt% of PVDF polymer without additives in Tamisolve^®^ NxG exhibited a spherulitic morphology with a pore size in the range of UF. Same pore size was also obtained for the MN2 membrane with PVP K17. The influence of PEG 200 without the presence of PVP K17 was also studied. PEG 200 at 20 wt% resulted to be the best concentration in the casting solution for its hydrophilic characters and for the high permeability of the membranes. Polymer concentration played also a crucial role in membrane morphology. Low polymer concentrations (10 wt%), for instance, resulted in a membrane structure characterized by macro-voids, large pore size and high permeability. The produced PVDF membranes showed a porosity ranging from 71% to 89% with a contact angle ranging from 75° to 91° for the top surface and from 78° to 117° for the bottom surface. The infrared spectroscopy (ATR-FTIR) confirmed the presence of both α and β phases of PVDF. The results of PWP as well as the filtration tests in terms of MB rejection and PRR confirm the potential use of these membranes in MF and UF applications for separation and purification processes. This work can also represent a good advance in the redesign of homopolymer PVDF membranes for their production using greener and more sustainable solvents.

## Figures and Tables

**Table 1:** Greener and conventional toxic aprotic solvents for the preparation of PVDF membranes.

Aprotic Dipolar Solvent	Polymer PVDF Type	Membrane Preparation Technique	Casting Conditions				Membrane Configurations	(Potential) Applications	Ref.
			Solutions Temp.	Casting Temp.	Casting Humidity	Coagulation Bath			
			(°C)	(°C)	(%RH)				
**Greener solvents**									
Tamisolve^®^ NxG	PVDFSolef^®^6010	NIPS	80–120	80–120	-	water	Flat sheet	UF/MF	In this work
	PVDFCopolymer Solef^®^21510	VIPS/NIPS	25	25	55	water	Flat sheet	membrane distillation (MD), Crystallization	[56,59]
	PVDF (MW: 543 000 Da)	NIPS	60	60	-	water	Flat sheet	supported PVDF NF-membranes for crosslinked PVDF	[60]
	PVDF (MW: 543 000 Da)	Spray-NIPS	100	100	-	water	Flat sheet	NF	[61]
Dimethyl sulfoxide (DMSO)	PVDF (Kynar740)	NIPS	80	80	-	water	Flat sheet	desalination by MD	[63]
	PVDFSolef^®^6012	Electrospinning	70	25	-	-	Nanofibers	water treatment	[54]
	PVDF (from Shanghai 3F)	NIPS	80	80	-	water	Flat sheet	MF	[64]
Triethylphosphate (TEP)	PVDFSolef^®^6010	VIPS/NIPS	100	100	-	water	Flat sheet	MF	[40]
	PVDF (Kynar740)	NIPS	80	80	-	water	Flat sheet	desalination by MD	[63]
	PVDFCopolymer Solef^®^21510	NIPS	25	25	-	water, water-ipa	Flat sheet	aqueous MD	[42]
	PVDF (from Shanghai 3F)	NIPS	80	80	-	water	Flat sheet	MF	[64]
Dimethyl isosorbide (DMI)	PVDFSolef^®^6010	VIPS/NIPS	120	25	65	water	Flat sheet	UF/MF	[39]
	PVDFSolef^®^6012	VIPS/NIPS	120	25	65	water	Flat sheet	UF/MF	
Polarclean^®^	PVDFSolef^®^1015	N-TIPS	130	-	-	water	Hollow fiber	water treatment	[65]
	PVDFSolef^®^1015	NIPS-TIPS	200	-	-	water	Flat sheet	water treatment	[66]
Cyrene^®^	PVDFSolef^®^6010	VIPS/NIPS	70	25	55	water	Flat sheet	UF/MF	[37]
Propylene carbonate	PVDF (MW: 170 000 Da)	TIPS ^1^	-	-	-	water	Hollow fiber	water treatment	[67]
Triacetin	PVDF	TIPS	170	170	-	water	Hollow fiber	membrane condenser	[68]
Ɣ-Butyrolactone	PVDF (MW: 170 000 Da)	TIPS	-	-	-	water	Hollow fiber	water treatment	[67]
**Conventional and toxic solvents**									
NMP	PVDFSolef^®^1015	N-TIPS	130	-	-	water	Hollow fiber	water treatment	[65]
	PVDF6010/P(VDF-co-HFP)	NIPS	60	60	-	Water/ethanol	Hollow fiber	DCMD	[69]
DMF	PVDF (from Shanghai 3F)	NIPS	80	80	80	water	Flat sheet	MF	[64]
	PVDFSolef^®^6012	Electrospinning	70	25	-	-	Nanofibers	MD	[70]
DMA	PVDF (from Shanghai 3F)	NIPS	80	80	-	water	Flat sheet	MF	[64]
Dibutyl phthalate (DBP)	PVDFSolef^®^1015	N-TIPS	130	130	-	water	Hollow fiber	water treatment	[65]

^1^ TIPS: thermal induced phase separation.

**Table 2:** Dope solutions compositions and investigated conditions to produce PVDF 6010 membranes.

Membrane Code	Casting Solution Composition				Temperature of Casting Solutions	Coagulation Conditions	
	PVDF 6010	PVP K17	PEG 200	TAM^®^		Temperature of Coagulation Bath	Time
	(wt%)	(wt%)	(wt%)	(wt%)	(°C)	(°C)	(minutes)
MN1	15	0	0	85	80	15	~8
MN2	15	5	0	85	80	15	~5
MN3	15	0	20	65	80	15	~5
MN4	15	0	40	65	120	15	~5
MN5	15	5	10	70	80	15	~5
MN6	15	5	15	65	80	15	~5
MN7	15	5	20	60	80	15	~5
MN8	15	5	40	40	120	15	~8
MN9	18	0	20	62	120	15	~8
MN10	18	5	20	57	120	15	~8
MN11	10	0	20	70	80	15	~5
MN12	10	5	20	65	80	15	~5

**Table 3:** Dynamic viscosity of solutions composition.

Membranes	Compositions	Viscosity
		(cP)
**MN1**	15 wt%PVDF-Tamisolve^®^ NxG	375.5 ± 1
**MN2**	15 wt%PVDF-5 wt% PVP K17-Tamisolve^®^ NxG	815.2 ± 1
**MN3**	15 wt%PVDF-20 wt% PEG_200_-Tamisolve^®^ NxG	787.3 ± 1
**MN5**	15 wt%PVDF-5 wt% PVP K17–10 wt%-PEG_200_- Tamisolve^®^ NxG	951 ± 1
**MN7**	15 wt%PVDF-5 wt% PVP K17–20 wt%-PEG_200_- Tamisolve^®^ NxG	1546 ± 1
**MN9**	18 wt%PVDF-20 wt% PEG_200_-Tamisolve^®^ NxG	921 ± 1
**MN11**	10 wt%PVDF-20 wt% PEG_200_-Tamisolve^®^ NxG	675 ± 1

**Table 4:** Thickness, porosity and contact angle results of the investigated membranes.

CODE	Thickness	Porosity	Contact Angle Air Side	Contact Angle Glass Side
	(µm)	(%)	(°)	(°)
MN1	100 ± 1	83 ± 2	91 ± 1	117 ± 1
MN2	125 ± 1	87 ± 2	77 ± 1	90 ± 1
MN3	104 ± 1	87 ± 1	76 ± 1	95 ± 1
MN4	107 ± 1	86 ± 1	88 ± 1	110 ± 1
MN5	123 ± 1	89 ± 2	86 ± 0	94 ± 1
MN6	122 ± 1	86 ± 1	76 ± 1	80 ± 1
MN7	109 ± 2	89 ± 1	75 ± 0	78 ± 1
MN8	110 ± 1	84 ± 2	85 ± 0	90 ± 1
MN9	133 ± 1	71 ± 1	84 ± 0	110 ± 0
MN10	138 ± 1	78 ± 1	82 ± 0	110 ± 0
MN11	90 ± 2	87 ± 1	86 ± 1	101 ± 1
MN12	96 ± 1	87 ± 1	79 ± 1	87 ± 1

**Table 5:** Filtration performance of PVDF membranes prepared with emerging greener and conventional solvents (concentration of MB may be different).

Polymer	Solvent	Solvent Toxicity ^1^	Additives	Foulant	PWP ^2^	Rejection	PRR	Ref.
PVDF (15 wt %)	Tamisolve^®^ NxG	P	PEG (20 wt%)	MB	257 L/m^2^hbar	57%	81%	In this work
PVDF (15 wt %)			PVP (5 wt%) PEG (20 wt%)	MB	269 L/m^2^hbar	86%	94%	In this work
PVDF (10 wt %)			PVP (5 wt%) PEG (20 wt%)	MB	88 L/m^2^hbar	79%	86%	In this work
PVDF (13 wt %)	TEP	P	PVP (3 wt%) PEG (24 wt%)	MB	2900 L/m^2^hbar	53%	-	[44]
PVDF/PMMA (12 wt %)	TEP	P	PVP (5 wt%) PEG (25 wt%)	MB	140 L/m^2^hbar	51%	-	[43]
PVDF (15 wt %)	DMAc	H	-	MB	-	40%	-	[94]
PVDF (18 wt %)	DMAc	H	-	MB	77 L/m^2^hbar	79%	94%	[93]
PVDF (15 wt %)	DMF	H	-	MB	5 L/m^2^hbar	40%	59%	[95]
PVDF (15 wt %)	DMF	H	PVP (1 wt%)	MB	-	45%	-	[97]
PVDF (20 wt %)	NMP	H	-	MB	1313 L/m^2^hbar	50%	-	[96]

^1^ Legend: P = more preferred, H = hazardous. ^2^ PWP: pure water permeability.

## Data Availability

Not Applicable.
